# Symptoms of Psychopathology in Hearing-Impaired Children

**DOI:** 10.1097/AUD.0000000000000147

**Published:** 2015-06-24

**Authors:** Stephanie C. P. M. Theunissen, Carolien Rieffe, Wim Soede, Jeroen J. Briaire, Lizet Ketelaar, Maartje Kouwenberg, Johan H. M. Frijns

**Affiliations:** 1 Department of Otorhinolaryngology and Head and Neck Surgery, Leiden University Medical Center, Leiden, The Netherlands; 2 Department of Developmental Psychology, Leiden University, Leiden, The Netherlands; 3 Dutch Foundation for the Deaf and Hard of Hearing Child, Amsterdam, The Netherlands; and 4 Leiden Institute for Brain and Cognition, Leiden, The Netherlands.

**Keywords:** Children, Cochlear implant, Hearing impairment, Mental health, Psychopathology

## Abstract

**Objectives:**

Children with hearing loss are at risk of developing psychopathology, which has detrimental consequences for academic and psychosocial functioning later in life. Yet, the causes of the extensive variability in outcomes are not fully understood. Therefore, the authors wanted to objectify symptoms of psychopathology in children with cochlear implants or hearing aids, and in normally hearing peers, and to identify various risk and protective factors.

**Design:**

The large sample (mean age = 11.8 years) included three subgroups with comparable age, gender, socioeconomic status, and nonverbal intelligence: 57 with cochlear implants, 75 with conventional hearing aids, and 129 children who were normally hearing. Psychopathology was assessed by means of self- and parent-report measures.

**Results:**

Children with cochlear implants showed similar levels of symptoms of psychopathology when compared with their normally hearing peers, but children with hearing aids had significantly higher levels of psychopathological symptoms, while their hearing losses were approximately 43 dB lower than those of children with implants. Type of device was related with internalizing symptoms but not with externalizing symptoms. Furthermore, lower age and sufficient language and communication skills predicted less psychopathological symptoms.

**Conclusions:**

Children who are deaf or profoundly hearing impaired and have cochlear implants have lower levels of psychopathological symptoms than children with moderate or severe hearing loss who have hearing aids. Most likely, it is not the type of hearing device but rather the intensity of the rehabilitation program that can account for this difference. This outcome has major consequences for the next generation of children with hearing loss because children with profound hearing impairment still have the potential to have levels of psychopathology that are comparable to children who are normally hearing.

## Abstract 2

Children with hearing loss are at risk for developing psychopathology, with detrimental consequences for academic and psychosocial functioning later in life. Yet the causes of the extensive variability in outcomes are not fully understood. This study showed that children who are deaf or profoundly hearing impaired and have cochlear implants have lower levels of psychopathological symptoms than children with moderate or severe hearing loss who have hearing aids. Most likely, it is not the type of hearing device, but rather the intensity of the rehabilitation program, that can account for this difference. Furthermore, lower age and sufficient language and communication skills predicted fewer psychopathological symptoms.

## INTRODUCTION

Bilateral permanent childhood hearing impairment affects approximately 1 to 1.3 of every 1000 live births (Fortnum & Davis 1997; Fortnum et al. 2001; Watkin & Baldwin 2011). This physical handicap influences communication and cognitive functioning but can also result in an increase in psychopathological symptoms (Hindley et al. 1994; Moeller 2007; Fellinger et al. 2012). Psychopathology refers to a broad spectrum of mental disorders. In the *Diagnostic and Statistical Manual of Mental Disorders, Fourth Edition*, a mental disorder is conceptualized as being a “clinically significant behavioral or psychological syndrome or pattern that occurs in an individual and that is associated with present distress, disability, a significantly increased risk of suffering death, pain, disability, or an important loss of freedom” (APA 2000). Symptoms of psychopathology during childhood can be divided into two categories: internalizing and externalizing symptoms. Internalizing symptoms are composed of depressive and anxious feelings, whereas externalizing symptoms refer to hyperactive, aggressive, and antisocial behavior (APA 2000). The prevalence of internalizing disorders in children with normal hearing is approximately 20% (Fleming et al. 1990; Fellinger et al. 2009b), while for externalizing disorders the rates range between 4% and 10% for hyperactive and aggressive behaviors (van Eldik et al. 2004; van Eldik 2005; Froehlich et al. 2007), and 1% and 3% for the more severe antisocial disorders (Maughan et al. 2004). Both internalizing and externalizing symptoms can have detrimental consequences on academic and psychosocial functioning later in life and are risk factors for other psychiatric disorders, as well as substance abuse (Hinshaw 1992; Birmaher et al. 1996; Kovacs & Devlin 1998; APA 2000; Zahn-Waxler et al. 2000; Lilienfeld 2003; Masten et al. 2005). In turn, those children who are affected, their families, and the society, are faced with increased mental health care costs and a high prevalence of school dropout rates (Reef 2010).

Children who are hearing impaired experience more internalizing and externalizing symptoms when compared with their normally hearing counterparts (King et al. 1989; van Eldik et al. 2004; van Eldik 2005; Konuk et al. 2006; Hintermair 2007; van Gent et al. 2007; Coll et al. 2009; Fellinger et al. 2012; Theunissen et al. 2011, 2012, 2013, 2014). The prevalence rates of various internalizing disorders in children who are hearing impaired are approximately 27% and that of externalizing disorders are 18%. These rates are based on both clinical interviews and medical records (van Gent et al. 2007; Fellinger et al. 2009b). However, there appears to be a lack of consistency within the literature with regard to the prevalence rates of specific disorders in children with hearing loss apart from depression (which has a reported lifetime prevalence of 26%; Fellinger et al. 2009b).

Various risk and protective factors, which typically affect the level of psychopathology, across those individuals within the population with hearing loss, have been identified. Children with significant hearing loss, who had higher levels of speech, language, or vocabulary, showed fewer symptoms of psychopathology (van Eldik et al. 2004; Percy-Smith et al. 2008; Barker et al. 2009; Theunissen et al. 2012). However, the use of sign language was related to more symptoms of psychopathology (van Gent et al. 2007; Remine & Brown 2010; Theunissen et al. 2011). Intellectual impairments were also related to more symptoms of psychopathology (van Eldik 2005; van Gent et al. 2007; Theunissen et al. 2014). Degree of hearing loss has often been believed to be of importance; however, the majority of literature has found no significant relation with children’s level of psychopathology (Wake et al. 2004; Fellinger et al. 2009b; Dammeyer 2010). Yet, early detection and intervention have been reported to be related to fewer symptoms of psychopathology (Theunissen et al. 2012). Furthermore, girls experience more internalizing symptoms (Vostanis et al. 1997; van Eldik 2005; van Eldik et al. 2004; Dammeyer 2010), whereas boys experience more externalizing symptoms (Theunissen et al. 2013). Mixed results have been obtained with regard to the relationship between psychopathology and socioeconomic status (SES), with some studies reporting no relationship (van Eldik et al. 2004; van Gent et al. 2007; Theunissen et al. 2012), whereas other studies have reported more symptoms of psychopathology in families with lower SES (Barker et al. 2009; Theunissen et al. 2014).

However, many of these studies did not use large or representative samples, which make it difficult to formulate concrete conclusions that are representative of the complete population with hearing loss. Yet, to be able to actually help this vulnerable group of children, we have to know which individuals are more at risk. In addition, identifying and understanding the causes of psychopathology will lead to an improvement in targeted screening, intervention, and counseling trajectories (Cosetti & Waltzman 2012). Therefore, the aim of this study was twofold: (1) to screen on and compare levels of internalizing and externalizing symptoms by using a multidimensional assessment, in three Dutch/Flemish groups: children with cochlear implants (CIs), children with conventional hearing aids, and children with normal hearing; and (2) to examine which risk and protective factors affect levels of psychopathological symptoms. Based on existing literature, it was expected that children with hearing loss would experience higher levels of psychopathology than their counterparts without any hearing loss and that sufficient language and communication skills would decrease these levels.

## MATERIALS AND METHODS

### Participants

In total, 261 children (mean age = 11.8, SD = 1.6) participated in this study. Table 1 shows characteristics of all the participants. The inclusion criteria stated that children must be living in the Netherlands or the Dutch-speaking part of Belgium (and that parent’s spoke Dutch with their child) and have a performance IQ of ≥80, which ruled out cognitive delays. For children with hearing loss, three more inclusion criteria were applied: (1) bilateral hearing loss of at least 40 dB in the least hearing-impaired ear; (2) which was pre- or perilingually detected; and (3) no other known comorbidities, such as visual impairment, hearing loss due to a syndrome (i.e., Treacher Collins or Usher syndrome), or autism spectrum disorders, because we wanted to examine a community sample.

**TABLE 1.**
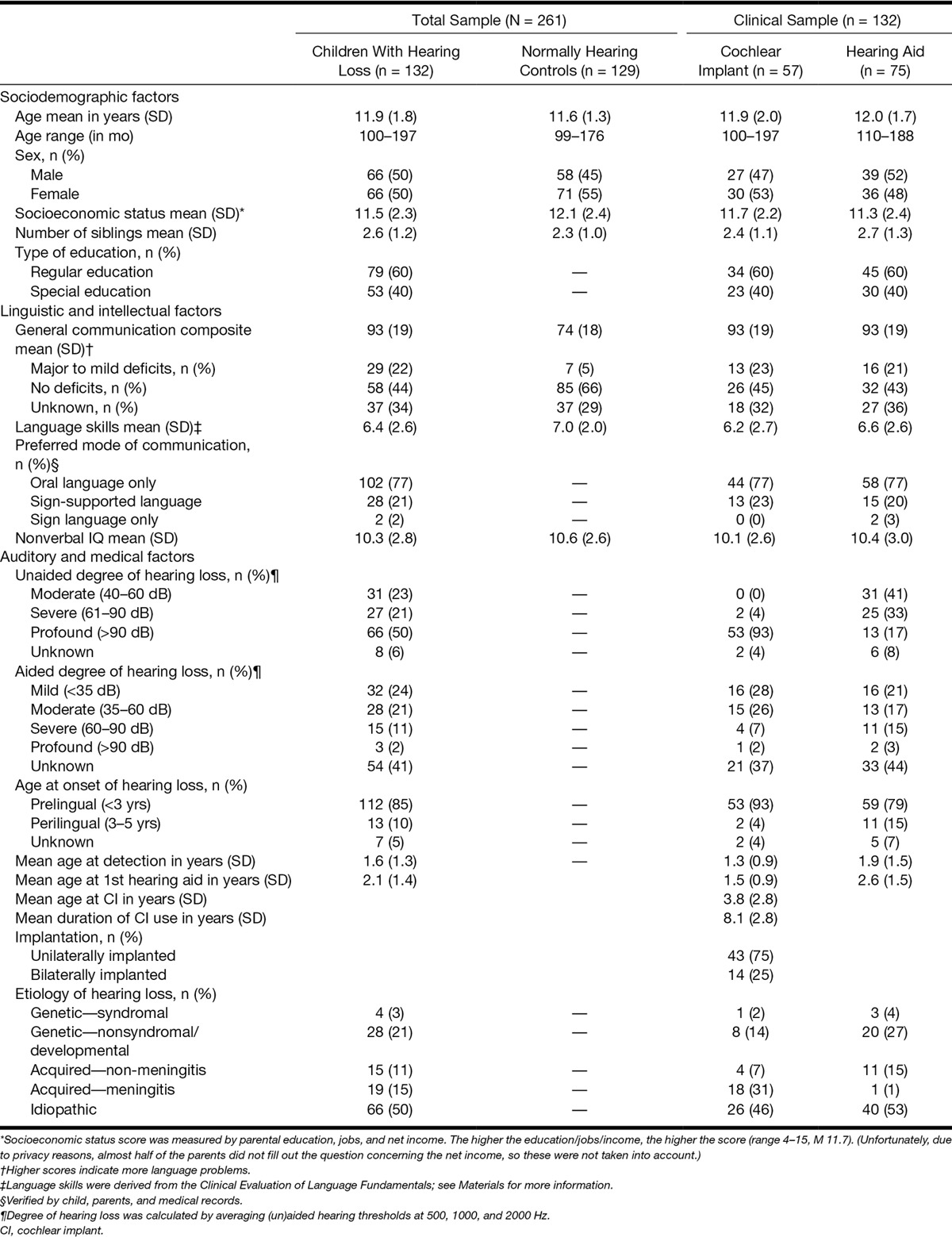
Characteristics of all participants

It is important to be aware of the differences in rehabilitation programs of children with CIs and children with hearing aids. CI recipients often follow an extensive and personalized rehabilitation program of approximately 1 year; children with hearing aids usually have much fewer moments of contact with professionals. Apart from the audiological fitting, most of these children only return to a hospital or speech and language center, when they actually have problems.

### Procedure

To collect a large Dutch/Flemish sample of children with hearing loss, we recruited our sample using various methods: in total, 28 special-needs schools (i.e., schools for children with hearing loss), 5 ambulatory care organizations (speech and hearing centers or residential schools), and 2 large academic hospitals were approached, of which 14 schools, 5 ambulatory organizations, and 2 hospitals agreed to participate. The other institutions refused for reasons related to time commitment. The total of the 21 participating institutions were an accurate reflection of the Dutch and Belgium system for children with hearing loss. In line with privacy policy, information packages and consent forms were sent to the parents of potential participants by the staff of the organizations, which agreed on participation, and in turn a signed consent form was sent back to the researchers. Therefore, the exact response rate is unknown. The controls were recruited at primary and secondary schools throughout the country, to reach a sociodemographically diverse sample. All participants were tested at their own homes or, when preferred, at their own school. This was carried out by one of the researchers. The participants were not financially compensated but instead received a small present after the testing session was finished.

All parents/caregivers gave consent for their child’s participation. Before actual data collection commenced, children were assured that their reactions would be processed anonymously and instructions were provided using the child’s preferred mode of communication. The participant could choose between two versions of assessment: the first version comprised written items exclusively, and in the second version each item was presented in written text and sign language simultaneously (by means of a video clip). All written or signed items were presented one by one on a laptop. Back translation of all signed items showed good convergence with the original items. A test session took approximately between 1 and 2 hours to complete, and the researcher communicated with the participant using their preferred mode of communication. Approval for the study was obtained by the Medical Ethics Committee of the Leiden University Medical Center under number P10.137.

### Materials

To optimally measure the level of psychopathological symptoms, various questionnaires were administered. All questionnaires were validated and standardized for the (normally hearing) population, except for the questionnaire measuring social anxiety. For social anxiety, a short index consisting of six items was developed especially for this study by a team of child psychologists, targeting the key aspects of social anxiety. All questionnaires had been administered to children with hearing loss and showed sufficient to good reliability, except for the questionnaire involving conduct disorder due to floor effects (Cronbach α 0.58 for the clinical sample; Theunissen et al. 2011, 2012, 2013; Kouwenberg et al. 2012). The higher the mean, the more symptoms are present.

The internalizing index consisted of questionnaires involving depressive symptoms, general and social anxiety, somatization, social phobia/obsessive compulsive disorder, and generalized anxiety disorder. For the externalizing index, questionnaires assessing the levels of aggression, delinquency, symptoms of psychopathy, oppositional defiant disorder, attention deficit hyperactivity disorder, and conduct disorder were included. We chose these specific areas, in line with the *Diagnostic and Statistical Manual of Mental Disorders, Fourth Edition* diagnoses, because they are among the most common psychopathological problems in childhood and can cause severe, pervasive impairments.

The majority of questionnaires were filled out by the children themselves, while some reports were completed by parents. The choice of respondent depended on which respondent was assumed to give the most appropriate and accurate answers. For example, internalizing symptoms are often better reported by children themselves because parents are known to underestimate the actual levels as such (Harris 1989; Fellinger et al. 2009b).

### Questionnaires Composing the Internalizing Index

#### Depression

The shortened version of the *Child Depression Inventory* (26 items) is a self-report, which assesses the presence of depressive symptoms in children aged 6 to 17 years (Kovacs 1985; Theunissen et al. 2011). An example item is “I feel lonely.”

#### Social Anxiety

For this study, child psychologists designed a new questionnaire (seven items), which measures the occurrence of different features of social anxiety (e.g., “I’m afraid of talking to someone I don’t know”) (Theunissen et al. 2012).

#### General Anxiety

The shortened version of the *Fear Survey Schedule for Children—Revised* (24 items) is a self-report, which measures the intensity of fears (e.g., of criticism, the unknown, small animals, danger, or death) in children from 7 to 17 years (Ollendick 1983).

#### Somatization

The *Somatic Complaint List* (11 items) examines the amount of self-reported physical symptoms in school-aged children (Jellesma et al. 2007). The reason for including this self-report is that internalizing symptoms in children can be expressed by somatic complaints only (Campo et al. 2004). An example item is as follows: “I have a stomach ache.”

#### Generalized Anxiety Disorder and Social Phobia/Obsessive Compulsive Disorder

The *Child Symptom Inventories* are parent-reported scales, which screen for emotional and behavioral disorders (Gadow & Sprafkin 1994; APA 2000). Only the two internalizing scales were used, assessing generalized anxiety disorder (seven items; e.g., “Has difficulty controlling worries”) and social phobia/obsessive compulsive disorder (three items; e.g., “Cannot get distressing thoughts out of his/her mind, for example, worries about germs or doing things perfectly”). Parents were asked how often these symptoms occurred.

### Questionnaires Composing the Externalizing Index

#### Aggression

In the *Self-Report Instrument for Reactive and Proactive Aggression* (36 items), participants were asked how often they performed several aggressive behaviors (e.g., kicking or arguing) in the last 4 weeks.

#### Delinquency

The self-report *Delinquency Questionnaire* (10 items) involves delinquent offences (e.g., shoplifting or stealing from parents; Baerveldt et al. 2003; Theunissen et al. 2013). Children were asked how many times they had committed these offences in the past year.

#### Psychopathy

The parent-completed *Psychopathy Screening Device* (20 items) reflects psychopathic behavior of the child (e.g., “Keeps his/her promises”; Frick et al. 1994). Parents were asked how often the behaviors occurred.

#### Behavioral Disorders

Three externalizing problems were derived from the *Child Symptom Inventories* (Gadow & Sprafkin 1994; APA 2000). The scales assessing attention deficit hyperactivity disorder (17 items; e.g., “Has difficulty paying attention to tasks or play activities”), oppositional defiant disorder (8 items; e.g., “Does things to deliberately annoy others”), and conduct disorder (15 items; e.g., “Has run away from home overnight”) were used.

### Composition of the Internalizing and Externalizing Indices

The Pearson correlations between all areas were computed to rule out large conceptual overlap. With correlations below 0.65, no collinearity appeared, implying that all areas contributed uniquely to the total index. Mean scores per area were calculated, which were standardized to eliminate scale differences between the questionnaires, using a mean of 100 (SD of 10), based on the normal-hearing group. With the standardized scores, two composite indices for internalizing and externalizing symptoms were computed. The indices had excellent internal consistencies, both with Cronbach αs of 0.91. Alpha’s retained their excellent values when examining responses of children with hearing loss exclusively.

### Language and Intelligence Tests

Nonverbal intelligence was obtained using two tests (*Block design* and *Picture arrangement*) of the *Wechsler Intelligence Scale for Children (WISC)—Third Edition* (Wechsler 1991; Kort et al. 2002). Age-equivalent norm scores based on Dutch standards (10 = average) were used to calculate one mean score. A random sampling (n = 23) across children with hearing loss who were assessed with a complete intelligence test earlier (either the Snijders-Oomen nonverbal intelligence test [Tellegen & Laros 1993] or the *WISC*) showed a high correlation between the scores of our tests and the IQ score (*r* = 0.79, *p* < 0.001). The tasks were not administered to 8 children with hearing loss and 17 children with normal hearing, due to time constraints.

Two tests (*Sentence comprehension* and *Story comprehension*) of the Dutch version of the *Clinical Evaluation of Language Fundamentals—Fourth Edition* (CELF) were administered (Semel et al. 1987; Kort et al. 2008). Norm scores were corrected for chronological age and one mean score was computed. When clinical or school records already contained CELF scores, which were no older than 2 years, these scores were used instead. The sentence comprehension task was not administered to 22 children with hearing loss and 16 controls, and the story comprehension task was not administered to 19 children with hearing loss and 16 controls, due to time constraints. The two children with hearing loss who used sign language exclusively received specific subtests of the *Assessment Instrument for Sign Language of the Netherlands* (Hermans et al. 2010). They both had sufficient sign language skills to interpret all questionnaires correctly.

The Dutch version of the *Children’s Communication Checklist version 2* was used to identify communication skills indicated by parents or caregivers (Bishop 1998; Geurts 2004). This questionnaire (with 70 items divided over eight scales) has been predominantly designed for assessing social and pragmatic language of children aged 4 to 16. The General Communication Composite (GCC) is conventionally obtained by using the scales Speech production, Syntax, Semantics, Coherence, Inappropriate initiation, Stereotyped conversation, Use of context, and Nonverbal communication. Each item could be scored from 0 (*never or less than 1 time a week*) to 3 (*several times a day or always*). The higher the GCC was the more communication deficits were present. Furthermore, the GCC was categorized by communication deficits (mild to major deficits: GCC ≥ 105, or no deficits: GCC ≤ 104). Around 30% of all parents did not fill out this questionnaire, and these nonresponders were equally spread over the three groups (CIs, hearing aids, or controls with normal hearing). Furthermore, age, sex, SES, language skills, and IQ also were equal between nonresponders and responders per group.

### Statistical Analyses

First, to give the reader a clearer picture of the data, all mean scores and percentages per group (that scored above 1 *SD* higher than the mean) for all individual questionnaires used in the composites are presented (see Appendix A, Supplemental Digital Content, http://links.lww.com/EANDH/A182). These data are not discussed further in detail because this article focuses on the internalizing and externalizing composite scores. The levels of the internalizing and externalizing symptoms between participants were compared using multivariate analysis of variance. Furthermore, Pearson correlations and regression analyses were carried out to examine risk and protective factors for psychopathology. When equal variances were not assumed between groups (using the Levene test), the corrected *p* value was used instead. Furthermore, although type of school can be an important factor, it is frequently the result of children’s functioning and not the cause, and this factor was therefore omitted from the analyses. The program SPSS version 21.0 was used. When a score (IQ or language) was not available, the participant was excluded from the analysis concerned.

## RESULTS

### Internalizing and Externalizing Symptoms

**Fig. 1.**
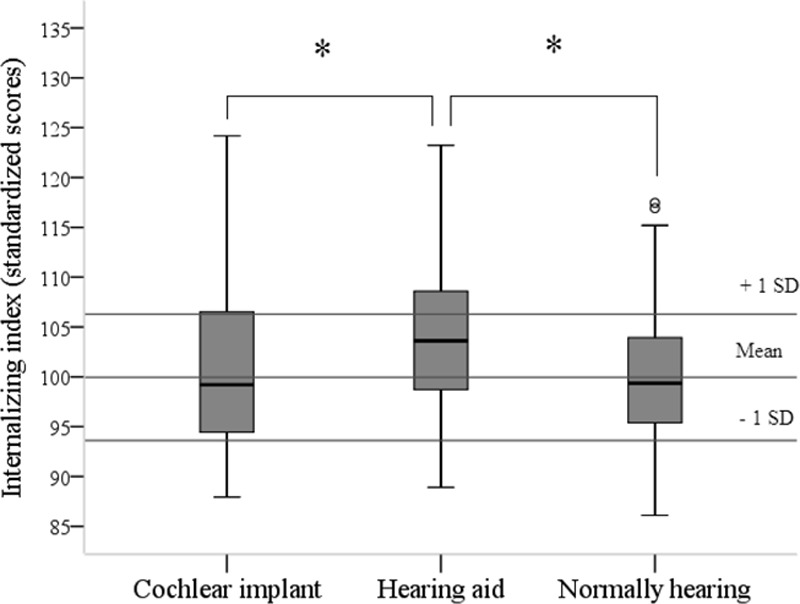
Internalizing index divided by group. *p < 0.05.

**Fig. 2.**
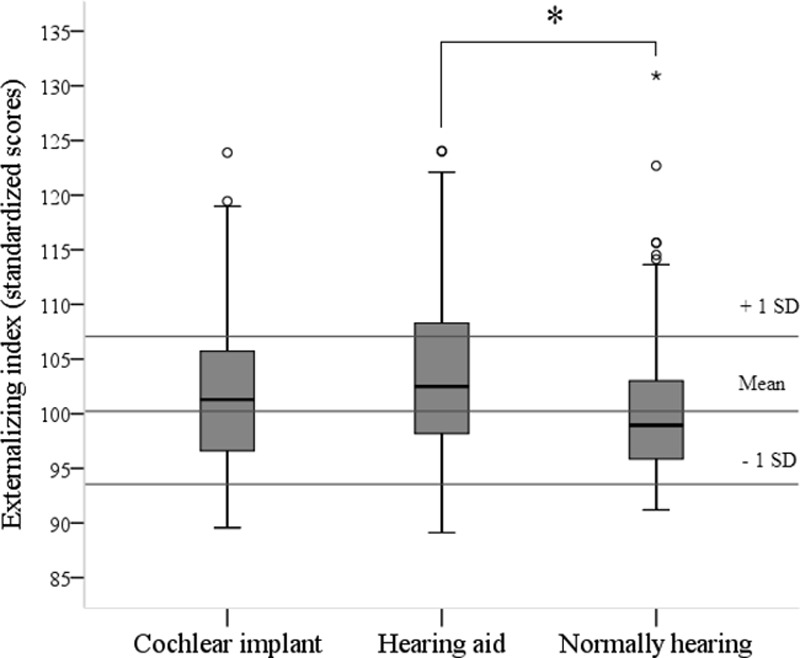
Externalizing index divided by group. *p < 0.05.

Children with CIs, children with hearing aids, and children with normal hearing were compared on the internalizing and externalizing indices (Figs. 1, 2). It first has to be said that the participants with hearing loss and with normal hearing were similar regarding age, gender, SES, and nonverbal intelligence. Yet, children with hearing loss exhibited lower language and communication skills than children who were normally hearing (language skills, Δ = 0.6 [95% confidence interval, 0.0–1.2] and communication skills, Δ = 19.3 [95% confidence interval, 13.8–24.7], respectively). Between children with CIs or with hearing aids, the above-mentioned variables were distributed equally.

A 3 (Group: CIs, hearing aids, controls) × 2 (internalizing or externalizing) multivariate analysis of variance revealed a multivariate effect for group, *F*(4,516) = 4.82, *p* < 0.001. Post hoc tests showed that CI recipients were not significantly different from children with normal hearing for both indices. Yet, children with hearing aids had significantly higher scores on both indices than children with normal hearing (internalizing, Δ = 3.6 [95% confidence interval, 1.7–5.5] and externalizing, Δ = 3.4 [95% confidence interval, 1.2–5.7]), meaning that the children with hearing aids experience more symptoms. In addition, children with hearing aids had significantly higher scores on the internalizing index than CI recipients (Δ = 2.8 [95% confidence interval, 0.4–5.3]).

When evaluating how many participants functioned 1 SD above the mean scores based on the normally hearing controls, we found that 26% of the CI recipients, 36% of the children with hearing aids, and 15% of the children with normal hearing scored above 1 SD for the internalizing index (*χ*^2^ (4) = 15.69, *p* < 0.004; Table 2). For the externalizing index, a statistical trend was found; 21% of the CI recipients, 29% of the children with hearing aids, and 12% of the controls had scores higher than 1 SD (*χ*^2^ (4) = 9.46, *p* = 0.052; Table 3). In addition, the group of children who scored above 2 SD for both indices were highest in the group of children with hearing aids (but due to low absolute numbers, no statistical tests were carried out). Furthermore, 4 (7%) children with CIs and 10 (13%) children with hearing aids scored 1 SD above the mean on both internalizing and externalizing indices.

**TABLE 2.**
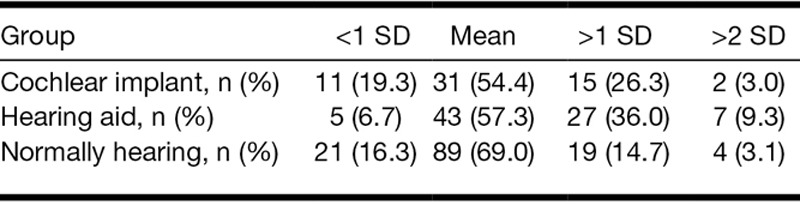
Distribution of internalizing indices

**TABLE 3.**
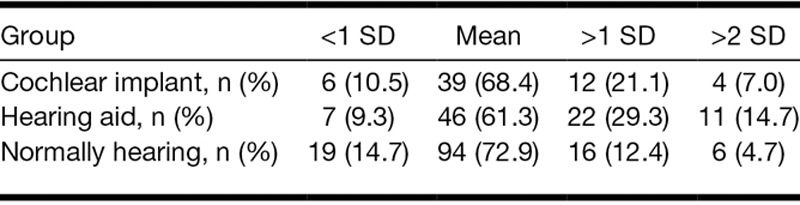
Distribution of externalizing indices

### Factors Associated With Psychopathological Symptoms

**Fig. 3.**
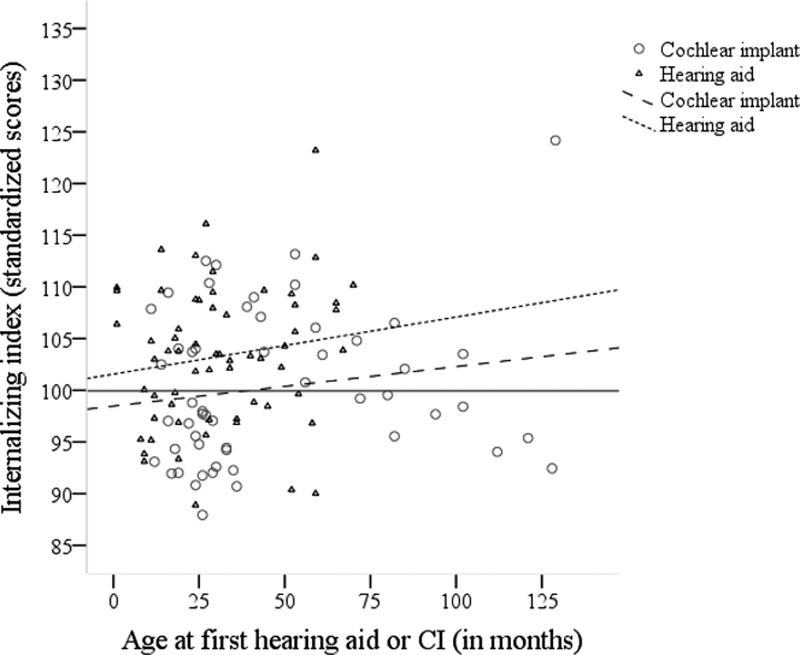
Internalizing index as function of age at intervention divided by type of device. CI, cochlear implant.

Table 4 shows which factors were investigated for the children with hearing loss (of which communication scores were available). Pearson correlations showed that better communication skills, better language skills, lower age at detection, lower age at intervention, and higher SES were significantly related to lower levels of internalizing symptoms. Note that for all children with hearing loss, age at intervention was the age at which the child received his/her first hearing aid, because every child with hearing loss starts with a 6-month trial of hearing aids due to potential maturation of the auditory system. To differentiate between children with hearing aids and implant recipients, age at first hearing aid or CI was plotted separately (Fig. 3). Irrespective of the age of amplification, children with hearing aids had higher internalizing indices than children who are normally hearing.

**TABLE 4.**
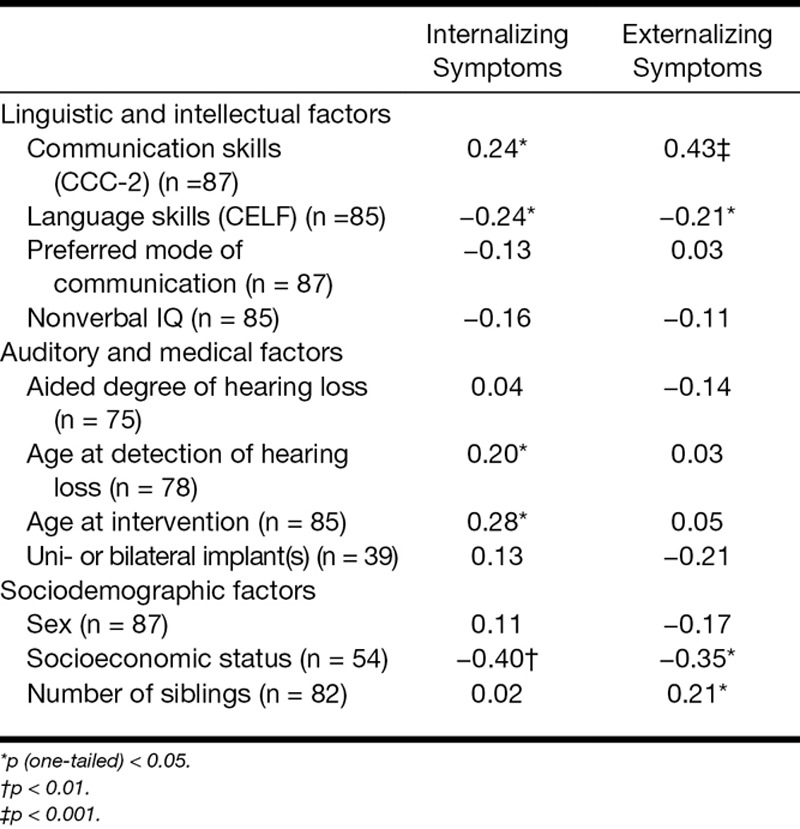
Pearson’s correlations for associated factors for psychopathology in children with hearing loss

When investigating the factors that affect the externalizing index, we found that fewer externalizing symptoms were related to better communication skills, language skills, and fewer siblings. Unaided degree of hearing loss was also tested, but due to redundant outcomes (i.e., the higher the degree, the less psychopathological symptoms, which is related to the fact that children with more severe losses received CIs), it was omitted from the results presented here.

### Influence of Type of Hearing Device on Symptoms of Psychopathology

To examine whether type of device had a direct impact on internalizing (Table 5) and externalizing (Table 6) symptoms, two hierarchical regression analyses were performed, while controlling for age, sex, and the significantly associated factors shown by Table 4. It was found that age, language skills, and type of hearing device contributed uniquely to the prediction of internalizing symptoms. The explained variance for this model was approximately 65% (*p* < 0.006). For externalizing symptoms, only communication skills contributed significantly. For this second model, the value of the explained variance reached 54% (*p* < 0.019).

**TABLE 5.**
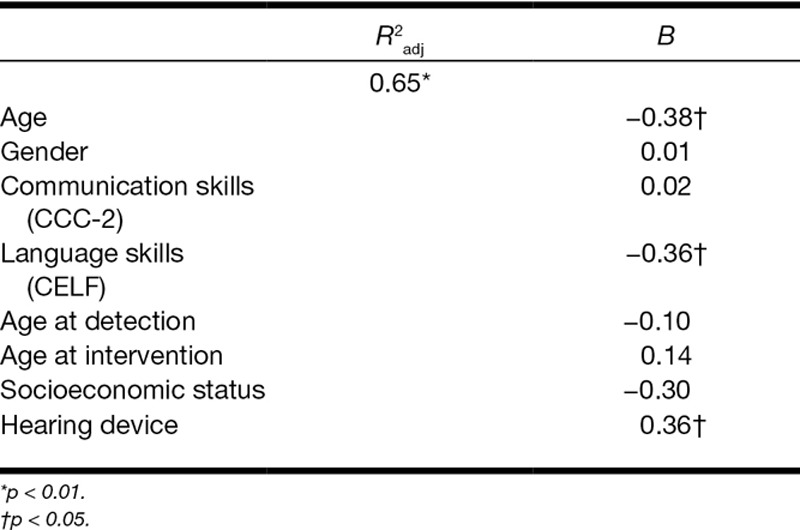
Hierarchical regression analysis for internalizing symptoms (n = 87)

**TABLE 6.**
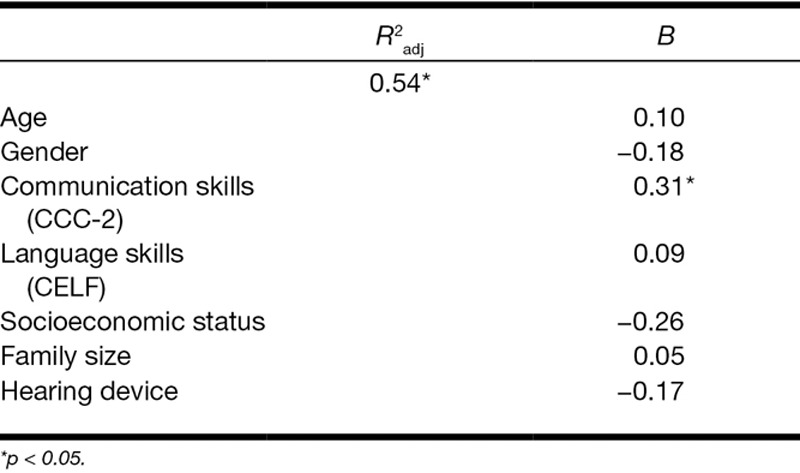
Hierarchical regression analysis for externalizing symptoms (n = 87)

## DISCUSSION

This study makes a novel contribution to the literature, by showing that children with CIs had lower levels of psychopathological symptoms than children with conventional hearing aids, despite the fact that these CI recipients experience more severe hearing loss. In fact, levels of psychopathology in children with CIs can be comparable to those of children with normal hearing. Type of device was related to internalizing symptoms but not to externalizing symptoms. In addition, lower age and sufficient language and communication skills were related to fewer symptoms of psychopathology. Furthermore, various associated factors for psychopathology were detected, including age at detection of hearing loss, age at intervention, SES, and number of siblings.

To the best of our knowledge, this study is one of the first in-depth, large-scale studies that investigated psychopathology in a multidimensional way, across three age- and gender-matched groups. Although past research has shown that hearing loss (van Eldik 2005; Hintermair 2007; Fellinger et al. 2012) and its associated factors (such as etiology, physical comorbidity, and communication problems; van Gent et al. 2012) have been associated with more internalizing and externalizing symptoms, the finding that not all children with hearing loss in general, but mainly those with hearing aids, are at risk of developing psychopathology, is new. Even with a major disadvantage involving degree of hearing loss (children with hearing aids had a mean hearing loss of 68 dB, while children with CIs had an approximate hearing loss of 111 dB), the hearing aided group reported more symptoms of psychopathology. In fact, children with hearing aids had higher scores on both indices for psychopathology than the other two groups.

When speculating what the causes for the strikingly positive outcomes for implanted children could be, we have to bear in mind that the factors which can affect the level of psychopathology were distributed similarly among both groups with hearing impairment, including age, sex, SES, nonverbal intelligence, language and communication skills, type of school, and mode of communication. Only age at detection and intervention differed, but we controlled for these factors. Yet, we should bear in mind that the group of children with CIs substantially differ from their hearing aided peers, when considering that they have received additional care upon the implantation of their device. Most CI recipients are enrolled in extensive CI rehabilitation programs, including speech therapy, family and child counseling, compared to children with hearing aids who receive no or almost no extra support apart from their hearing aid. It can also be possible that although language is not different, children with hearing aids may have trouble communicating in noising environments or with peers.

In addition, parents of children with CIs might have higher expectations after implantation and encourage and stimulate their child more. It could be hypothesized that when children with hearing aids underwent similar rehabilitation programs, that they would also have had levels of psychopathological symptoms, which equaled those of children with normal hearing, similar to the CI recipients. A follow-up study design could perhaps provide the opportunity to draw firmer conclusions on causality. In addition, it should be noted that many more factors could be relevant for the development of psychopathology. For example, concomitant handicaps, parent-child attachment, or intrapersonal factors could be contributive in this respect.

The negative effects found for children with hearing loss can be reduced by adequate language and communication skills. Our study confirmed the findings that better oral communication skills are related to lower levels of psychopathology (van Eldik et al. 2004; Percy-Smith et al. 2008; Barker et al. 2009; Fellinger et al. 2009a;Stevenson et al. 2010). These findings stress the fact that social language, pragmatic language, and communication are of utmost importance for preventing psychopathology. Acceptance of hearing peers would also be more likely when communication skills are good (Bat-Chava & Deignan 2001). In addition, better communication skills increase the chance that children with hearing loss attend regular schools, where they will meet and interact with more children who have normal hearing, which would even further improve their social interactions and communication skills. Hence, parents and professionals who work with children with hearing loss should focus on and encourage well-developed and age-appropriate communication skills.

Currently, many children with hearing loss with psychopathology receive no treatment, and only the ones which evidently stagnate in their development are referred to specialized care. This is underlined by the fact that only a minority of children with psychopathology (approximately 25%), whom are without any hearing impairment, receive mental health care services (Tuma 1989; Zahner et al. 1992; Vernon & Leigh 2007; Ford et al. 2008). It is assumed that because of the language barrier and subsequent isolation, relatively more children with hearing loss do not have access to mental health services (Cabral et al. 2012; Fellinger et al. 2012). Therefore, we must use a multidisciplinary approach not only for implanted children but also for this group and include speech therapists and child and family counselors in a way which follows a particular protocol. This way, we can focus on early detection of additional problems and proactively approach the children with hearing loss to screen them on symptoms of psychopathology.

Some limitations existed within this study. First, although participation was completely voluntary, it could be plausible to posit that the more active children or the children who were more interested in societal concerns or deaf culture enrolled more often, resulting in a potential selection bias. Second, the exact response rate (i.e., the amount of nonresponders) was unknown, due to the privacy policy of the participating institutions, making it impossible to compare characteristics of responders with nonresponders. Third, when a variable was missing, the participant was excluded from the analysis concerned. Yet, the group of children with missing data was not different (on age, sex, SES, language skills, and IQ) than the ones with complete data sets.

In conclusion, despite significantly less severe hearing loss, children with hearing aids have higher levels of psychopathological symptoms than CI recipients. Children with CIs had similar levels of psychopathological symptoms when compared with the group of children without any hearing loss. Further research involving the pathways leading to these differences between children with CIs and hearing aids is needed.

## ACKNOWLEDGMENTS

We thank all children and their parents for participation in this study.

## Supplementary Material
