# Supplementary material for: Symptoms of Psychopathology in Hearing-Impaired Children
Source: Ear Hear. 2015 Jun 24;36(4):e190–8. doi: 10.1097/AUD.0000000000000147 (PMC4478069; doi:10.1097/AUD.0000000000000147)
Supplement: Supplementary file 1 [file aud-36-e190-s001.docx]

Appendix A.

*Scores of individual questionnaires*

|  | Mean | | | | > 1 *SD* (%) | | |
| --- | --- | --- | --- | --- | --- | --- | --- |
|  | Cochlear implant | Hearing aid | Normally hearing | *F* (by ANOVA) | Cochlear implant | Hearing aid | Normally hearing |
| **Internalizing index** |  |  |  |  |  |  |  |
| Depression | 100.8 | 101.9 | 100 | 0.79 | 14.0 | 23.0 | 13.2 |
| Social anxiety | 99.5 ^a, b^ | 103.5 ^b^ | 100 ^a^ | 3.63* | 12.5 | 15.5 | 16.4 |
| General anxiety | 100.1 | 102.2 | 100 | 1.15 | 12.5 | 15.5 | 14.1 |
| Somatization | 99.2 | 102.1 | 100 | 1.50 | 15.8 | 17.3 | 18.8 |
| Generalized anxiety disorder | 103.1 ^a, b^ | 105.6 ^b^ | 100 ^a^ | 4.20* | 27.7 ^a, b^ | 34.4 ^a^ | 19.8 ^b^ |
| Social phobia / Obsessive compulsive disorder | 101.9 | 105.2 | 100 | 3.04 | 17.0 ^a, b^ | 28.1 ^a^ | 14.0 ^b^ |
| **Externalizing index** |  |  |  |  |  |  |  |
| Aggression | 98.9 | 103.4 | 100 | 2.91 | 12.5 ^a, b^ | 27.8 ^a^ | 14.1 ^b^ |
| Delinquency | 96.6 | 99.6 | 100 | 2.82 | 8.8 | 9.3 | 10.1 |
| Psychopathy | 104.5 | 103.3 | 100 | 2.69 | 31.9 | 27.0 | 17.3 |
| ADHD | 104.7 ^a^ | 103.4 ^a, b^ | 100 ^b^ | 4.52* | 25.5 ^a^ | 21.9 ^a, b^ | 11.6 ^b^ |
| ODD | 105.5 ^a^ | 102.9 ^a, b^ | 100 ^b^ | 4.60* | 27.7 | 21.9 | 16.3 |
| CD | 106.7 ^a, b^ | 106.8 ^b^ | 100 ^a^ | 4.72* | 25.5 ^a^ | 23.4 ^a^ | 10.5 ^b^ |

*Note.* In case of a significant difference between the mean scores (by ANOVA), post hoc *t*-tests were performed and letter-superscripts indicate differences at *p* < .05. In case of a significant difference between the *SD* scores (by Chi-square tests), letter-superscripts indicate differences at *p* < .05.

**p* < .05.
